# Rapid Differential Diagnosis between Extrapulmonary Tuberculosis and Focal Complications of Brucellosis Using a Multiplex Real-Time PCR Assay

**DOI:** 10.1371/journal.pone.0004526

**Published:** 2009-02-19

**Authors:** María Isabel Queipo-Ortuño, Juan D. Colmenero, Pilar Bermudez, María José Bravo, Pilar Morata

**Affiliations:** 1 Biochemistry and Molecular Biology Department, Faculty of Medicine, University of Malaga, Malaga, Spain; 2 CIBER Fisiopatología Obesidad y Nutrición (CB06/03) Instituto de Salud Carlos III, Madrid, Spain; 3 Infectious Diseases Service, Carlos Haya University Hospital, Malaga, Spain; 4 Microbiology Service, Carlos Haya University Hospital, Malaga, Spain; 5 Immunology Service, Carlos Haya University Hospital, Malaga, Spain; Columbia University, United States of America

## Abstract

**Background:**

Arduous to differ clinically, extrapulmonary tuberculosis and focal complications of brucellosis remain important causes of morbidity and mortality in many countries. We developed and applied a multiplex real-time PCR assay (M RT-PCR) for the simultaneous detection of *Mycobacterium tuberculosis* complex and *Brucella* spp.

**Methodology:**

Conventional microbiological techniques and M RT-PCR for *M. tuberculosis* complex and *Brucella* spp were performed on 45 clinical specimens from patients with focal complications of brucellosis or extrapulmonary tuberculosis and 26 control samples. Fragments of 207 bp and 164 bp from the conserved region of the genes coding for an immunogenic membrane protein of 31 kDa of *B. abortus* (BCSP31) and the intergenic region SenX3-RegX3 were used for the identification of *Brucella* and *M. tuberculosis* complex, respectively.

**Conclusions:**

The detection limit of the M RT-PCR was 2 genomes per reaction for both pathogens and the intra- and inter-assay coefficients of variation were 0.44% and 0.93% for *Brucella* and 0.58% and 1.12% for *Mycobacterium*. M RT-PCR correctly identified 42 of the 45 samples from patients with tuberculosis or brucellosis and was negative in all the controls. Thus, the overall sensitivity, specificity, PPV and NPV values of the M RT PCR assay were 93.3%, 100%, 100% and 89.7%, respectively, with an accuracy of 95.8% (95% CI, 91.1%–100%). Since M RT-PCR is highly reproducible and more rapid and sensitive than conventional microbiological tests, this technique could be a promising and practical approach for the differential diagnosis between extrapulmonary tuberculosis and focal complications of brucellosis.

## Introduction

The global burden of tuberculosis remains enormous and brucellosis continues to be the most common zoonotic infection worldwide, representing a major source of human disease [1]–[2]. Both tuberculosis and brucellosis are granulomatous diseases with great clinical polymorphism. Extrapulmonary forms account for 10% to 40% of all cases of tuberculosis [3]–[4] and focal complications are present in 20% to 40% of brucellosis patients [5]–[6]. Extrapulmonary tuberculosis and focal forms of brucellosis have been described in almost all organs and systems, with osteoarticular, genitourinary, hepatic or central nervous system involvement being frequent in both diseases [4]–[5]. Accordingly, in areas of high incidence, a differential diagnosis between both processes is often necessary [7].

Culture remains the gold standard for the diagnosis of tuberculosis and brucellosis. However, as both *Brucella* spp and *Mycobacterium tuberculosis* are slowly growing pathogens, cultures are labor intensive, which can at times lead to unacceptable delays in diagnosis. Furthermore, cultures can be very insensitive in some cases of extrapulmonary tuberculosis and focal forms of brucellosis [8]–[9]. To overcome certain limitations of conventional microbiological techniques, PCR-based assays may be useful for the diagnosis of both tuberculosis and human brucellosis [10]–[11]. The use of real-time PCR technology reduces the time to identification of bacterial DNA directly from clinical samples. Additionally, considerable time and effort can be saved by simultaneously amplifying multiple sequences in a single reaction. This strategy, named Multiplex PCR, has proven to be very useful in different clinical scenarios [12]–[13].

The aim of this study was to develop a multiplex real-time PCR (M RT-PCR) assay to simultaneously detect *Brucella* spp and *M. tuberculosis* complex DNA and analyze its yield in the rapid differential diagnosis between extrapulmonary tuberculosis and certain focal complications of brucellosis.

## Methods

### Bacteria species and strains

The strains of *Brucella* used in this study were supplied by the Microbiology Department of the Faculty of Medicine at Valladolid University, except for the vaccine strains B-19 and Rev-1, kindly provided by the Agriculture Department of the Andalusian Regional Government. These strains were cultured on *Brucella* agar (Difco, USA) and incubated at 37°C with 5% CO_2_ for 48 h. All non-tuberculous mycobacteria strains except *M. avium* and *M. celatum* were provided by the Colección Española de Cultivos Tipo (CECT) (Valencia, Spain). These strains were grown on Lowenstein-Jensen medium (Biomedics, Spain) at 37°C for 2–4 weeks.

### Study population

Forty-five non-blood clinical specimens from 25 patients with different focal complications of brucellosis and 18 patients with extrapulmonary tuberculosis were studied by M RT-PCR assay. One brucellosis patient who had two different focal complications and another with tuberculous spondylitis and therapeutic failure each provided two different samples.

The samples came from vertebral or other bone tissue (13 patients), lymph nodes (7 patients), tissue or pus from hepatosplenic abscesses (6 patients), cerebrospinal fluid (CSF) (5 patients), synovial fluid (4 patients), urine or kidney tissue (4 patients), pleural fluid (3 patients), pericardial tissue (2 patients) and thyroid tissue (1 patient).

Control samples were obtained from 26 patients with other disorders initially involving a differential diagnosis with extrapulmonary tuberculosis or brucellosis: pyogenic hepatosplenic abscesses (7 patients), septic arthritis (6 patients), pyogenic vertebral osteomyelitis (6 patients), bacterial meningitis (3 patients) and non-tuberculous vertebral osteomyelitis, hepatic Hodgkin lymphoma, kidney abscess and pyogenic pericarditis (1 each).

The diagnosis of brucellosis was established according to one of the following criteria: first, isolation of *Brucella* spp. from blood or any other body fluid or tissue sample or second, the presence of a compatible clinical picture together with the demonstration of specific antibodies at significant titers or seroconversion. Significant titers were considered to be a standard tube agglutination test (SAT) titer of ≥1/160 or an immunocapture agglutination test ≥1/320. The diagnosis of tuberculosis was based on isolation of *M. tuberculosis* or the presence of caseating granulomas, with or without acid-fast bacilli, in a patient with a compatible clinical picture and good therapeutic response to antituberculous treatment.

#### Ethics Statement

All patients provided written informed consent prior to the collection of biological samples. The utilization of samples for research purposes was approved by the Ethical Committee of Carlos Haya University Hospital, Malaga, Spain.

### Microbiological studies

Two blood cultures were performed for all patients with suspected brucellosis. Blood samples were incubated in a non-radiometric semiautomatic BACTEC 9240 system (Becton Dickinson, Diagnostic Instrument Systems, Sparks, MD), and processed according to usual techniques. All isolates were identified according to normalized protocols [14].

All non-blood samples were stained with Gram, Ziehl–Neelsen and auramine and cultured onto blood and chocolate agar media, MacConkey agar, *Brucella* agar Lowenstein–Jensen and/or Middlebrook medium (BACTEC MGIT 960, Becton Dickinson, Diagnostic Systems, Spark, MD). SAT was performed as described [15] and immunocapture-agglutination test (Brucellacapt; Vircell SL, Sante Fé, Granada) was done following the manufacturer's instructions [16].

### DNA extraction

All samples destined for M RT-PCR were maintained at −20°C until processing. The volume varied depending on the type of sample. DNA was extracted using the UltraClean Tissue DNA isolation Kit (Mo Bio Laboratories). Prior to DNA extraction, homogenized samples from the different tissues, CSF, synovial or pleural fluid and purulent sample collections were resuspended in 1 ml of molecular biology water, mixed and centrifuged at 15000×g for 5 min. The supernatant was discarded and the pellet was resuspended with the volume of buffer outlined in the manufacturer's instructions. DNA pellets were resuspended in 50 µl molecular biology water and stored at 4°C until use. Aliquots of 5 µl of the suspension (template DNA) were used for PCR analysis. To monitor contamination, negative controls were included during each DNA extraction procedure.

### Real-time PCR primers and probes

For the detection of *Brucella* spp, a 207 bp fragment from the conserved region of the gene which encodes an immunogenic membrane protein of 31 kDa of *B. abortus* (BCSP31) specific to the *Brucella* genus and present in all its biovars was amplified using the primers B1 and B2. Primers M1 and M3 amplifying a sequence of 164 bp based on the intergenic region of the genes coding for a mycobacterial two-component system SenX3-RegX3 were used for the identification of *M. tuberculosis* complex. This DNA target sequence is present in all strains of *M. tuberculosis* complex and is absent from all other non-tuberculous mycobacterial strains. The sequences and positions of the amplification primers and the detection probes are shown in Table 1. Fluorescent hybridization probes were designed to anneal within the gene fragment generated by amplification of the corresponding target. The *Brucella* hybridization probe set (SB1 and SB2) was fluorescein- and LCRed640-labeled and the *M. tuberculosis* complex hybridization probe set (SM1 and SM3) was fluorescein-and LCRed705-labeled. An extensive search of several databases, including EMBL and GenBank databases, indicated that neither the primers nor the probes shared significant homology with any known nucleotide sequence. All primers and probes were synthesized by Proligo (Sigma Aldrich).

**Table 1:** Nucleotide sequences and positions of primers and probes for amplification and detection of *Brucella* spp. and *M. tuberculosis* complex for M RT-PCR.

Oligonucleotide	Sequence	Position	Product Length (bp)
B1 up	5′-ggctcggttgccaatatcaat-3′	788-810	
B2 down	5′-gtctgcgaccgatttgatgt-3′	995-977	207
SB1 FL probe	5′-aggcaacgtctgactgcgtaaagcc -FL-3′	862-838	
SB2 Red probe	5′-Red 640 -actccagagcgcccgacttgatcg-Phos-3′	835-812	
M1 up	5′-cggctaatcacgacggcac -3′	1114-1132	
M3 down	5′-ctcttcctctcgttgtgacctgtt-3′	1277-1254	164
SM1 FL probe	5′-tggctcttccggcgttgatcgag- FL-3′	1177-1199	
SM3 Red probe	5′-Red 705-cctatcacgacgacgagcgacccga-Phos-3′	1225-1201	

Red 640- LightCycler Red 640, Red 705- LightCycler Red 705, FL-5,6-carboxifluoresceína.

### Real-time PCR assay conditions

Amplification and melt curve analysis were performed using a LightCycler (Roche Diagnostics, Indianapolis, IN). Reactions were carried out in a total volume of 20 µl. PCR mixes contained primers and probes at final concentrations of 0.6 µM and 0.2 µM, respectively. FastStart DNA Master Hybridization Probes kit (Roche Molecular Biochemicals, Mannheim, Germany) was used; 4 µl of the master mixture and 5 µl of DNA samples were loaded into glass capillary tubes. After a short centrifugation (3000×g for 10 s) the sealed capillaries were placed into the LightCycler rotor. After an initial polymerase activation and denaturation step at 95°C for 10 min, the samples ran 45 amplification cycles, each comprising denaturation (95°C for 10 s), annealing (60°C for 20 s), and extension (72°C for 10 s) in the LightCycler 2.0, with a temperature transition rate of 20°C/s for all steps. After completion, a melting curve was recorded by heating to 95°C for 0 s at 20°C/s, holding at 41°C for 20 s at 20°C/s, and then heating slowly at 0.1°C/s until 85°C. A final cooling step of 40°C for 15 s was added. The peak melting temperature obtained represented the specific amplified product. Each product was tested using different fluorometer channels. The dye signal generated by the BCSP31 product was measured at 640 nm and the SenX3-RegX3 intergenic region product signal was measured at 705 nm following 60°C, 20 s annealing incubation.

A compensation data file was created using the LightCycler-Color Compensation Set (Roche Diagnostic, Indianapolis, IN). Fluorescence curves were analyzed with the LightCycler software v. 4.0. To minimize experimental variability, we used the automated second derivate maximum estimation method to determine the amplification crossing point (Cp or threshold cycle) that marked the cycle at which fluorescence of the sample became significantly different from the baseline signal.

Positive controls, included in all tests, comprised serial dilutions of *B. abortus* B-19 and *M. tuberculosis* DNA. Negative controls were also included and contained all the elements of the reaction mixture except template DNA. To guarantee the reliability of the results, all samples were processed in duplicate. A sample was defined as positive only when the Cp value was ≤36 cycles in both replicates and the melting temperature peak was consistent with that produced by the corresponding positive controls. The absence of an amplification curve or a Cp value ≥37 cycles was considered to indicate a negative sample.

Universal precautions and one-way flow of DNA extraction and amplification were used to prevent contamination. To avoid potential observer bias, the status of each patient for *Brucella* and *Mycobacterium* infection was unknown during the PCR assay.

### Sequencing of M RT-PCR product

To confirm the identities of the amplified fragments, the M RT-PCR products for *Brucella* spp and *M. tuberculosis* complex were sequenced. The ABI PRISM Big Dye Terminator Cycle sequencing reaction kit v. 3.0 (Applied Biosystems, Madrid, Spain) was used for the sequencing reactions. Sequence analysis was by capillary electrophoresis in an ABI PRISM, model 3100 automated sequencer (Applied Biosystems).

### Statistical Analysis

Sensitivity, specificity, positive and negative predictive values, accuracy, likelihood ratios (LR) and 95% confidence intervals (CI) were calculated using the Twobytwo 1.0 analyzer program.

## Results

### Analytical sensitivity

The M RT-PCR analytical sensitivity was initially determined by amplifying ten-fold serial dilutions of DNA from *B. abortus* B-19 and *M. caprae*. The detection limit was 2×10^0^ genomes per reaction for both pathogens. The assays showed a linear quantitative range over five orders of magnitude from 2×10^5^ down to 2×10^0^ genomes per reaction, with linear regression equations of Cp = −3.37 log (genome number.) +36.92 and Cp = −3.32 log (genome number.) +36.89, correlation coefficients (R^2^) values of 0.99 and a PCR efficiencies (E) of 2.0 for *Brucella* and *Mycobacterium* respectively.

### Reproducibility

Intra-assay variability was determined by amplifying, in quadruplicate, dilutions of DNA from *B. abortus* B-19 and *M. caprae*, equivalent to 2×10^4^ to 2×10^0^ copies per reaction. Threshold cycle values (Cp) obtained for the same dilutions on five different days were used to determine the inter-assay variability. The mean coefficients of variation (CV) for intra-assay repetitions were 0.44% for *Brucella* and 0.58% for *M. tuberculosis complex*, with CV values of 0.32%, 0.74%, 0.35%, 0.35% and 0.35%, 0.63%, 0.65%, 0.69% for the samples with 2×10^4^, 2×10^3^, 2×10^1^, 2×10^0^ copies per reaction of *Brucella* and *Mycobacterium*, respectively. The mean inter-assay CVs for the whole group of samples were 0.93% for *Brucella* and 1.12% for *Mycobacterium* with CV values of 0.73%, 0.80% 0.66%, 1.14% and 1.09%, 0.91%, 1.21%, 1.29% for the samples with 2×10^4^, 2×10^3^, 2×10^1^, 2×10^0^ copies per reaction of *Brucella* and *Mycobacterium*, respectively.

### Specificity

**Figure 1:**
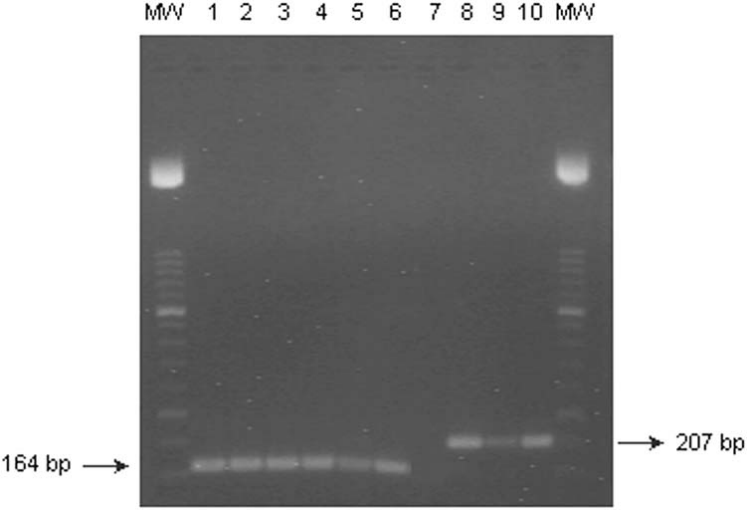
Agarose gel electrophoresis and ethidium bromide staining. Lanes: MW, molecular size DNA ladder XIII; 1, positive control (*M. caprae*); 2 to 3, DNA from two strains of *M. tuberculosis* complex (*M. tuberculosis* and *M. africanum*); 4 to 6, samples of pleural fluid, psoas abscess and urine, respectively, from three patients with tuberculosis; 7, no ADN added; 8 positive control (*B. Melitensis Rev-1*); 9 to 10, DNA from hepatic abscess and kidney tissue, respectively, from two patients with brucellosis.

To confirm the specificity, we tested different strains of *Brucella*, *M. tuberculosis* complex and non-tuberculous Mycobacteria (Table 2). Only amplified fragments from *M. tuberculosis* complex were detected at a wavelength of 705 nm, indicating the fluorogenic probes were specific for *M. tuberculosis* complex and did not cross-react with *Brucella* spp or other non-tuberculous Mycobacteria strains. Fluorescent signals at 640 nm were obtained with all strains of *Brucella* spp assayed but not with any *Mycobacterium* strain (Figure 1). The M RT-PCR assay was therefore 100% specific.

**Table 2:** Multiplex PCR results with DNA from different *Brucella* and *Mycobacterium* strains.

*Species*	*Biovar*	*Strain*	*Origin*	*M tuberculosis complex*	*Brucella*
*Brucella melitensis*	1	16 M	FMV	**−**	**+**
*Brucella melitensis*	1	Rev 1	CAJA	**−**	**+**
*Brucella melitensis*	2	63/9	FMV	**−**	**+**
*Brucella melitensis*	3	Ether	FMV	**−**	**+**
*Brucella melitensis*	2	(clinical strain)	FMV	**−**	**+**
*Brucella melitensis*	3	(clinical strain)	FMV	**−**	**+**
*Brucella abortus*	1	(clinical strain)	FMV	**−**	**+**
*Brucella abortus*	1	B19	CAJA	**−**	**+**
*Brucella abortus*	2	86/8/59	FMV	**−**	**+**
*Brucella abortus*	3	Tulya	FMV	**−**	**+**
*Brucella abortus*	4	292	FMV	**−**	**+**
*Brucella abortus*	5	B3196	FMV	**−**	**+**
*Brucella abortus*	6	870	FMV	**−**	**+**
*Brucella abortus*	7	63/75	FMV	**−**	**+**
*Brucella abortus*	9	C/68	FMV	**−**	**+**
*Brucella suis*	1	10036	FMV	**−**	**+**
*Brucella suis*	2	10510	FMV	**−**	**+**
*Brucella suis*	3	10511	FMV	**−**	**+**
*Brucella suis*	4	40	FMV	**−**	**+**
*Brucella suis*	5	10980	FMV	**−**	**+**
*Brucella neotomae*		10084	FMV	**−**	**+**
*Brucella ovis*		Reo198	FMV	**−**	**+**
*Brucella canis*		10854	FMV	**−**	**+**
*Mycobacterium tuberculosis*		1100	HCH	**+**	**−**
*Mycobacterium caprae*		1040	HCH	**+**	**−**
*Mycobacterium africanum*		1031	HCH	**+**	**−**
*Mycobacterium bovis*		530	HCH	**+**	**−**
*Mycobacterium avium*		1062	ATCC	**−**	**−**
*Mycobacterium xenopi*		1071	HCH	**−**	**−**
*Mycobacterium kansasii*		1085	HCH	**−**	**−**
*Mycobacterium chelonae*		1052	HCH	**−**	**−**
*Mycobacterium gordonae*		953	HCH	**−**	**−**
*Mycobacterium fortuitum*		944	HCH	**−**	**−**
*Mycobacterium scrofulaceum*		702	HCH	**−**	**−**
*Mycobacterium szulgai*		CC 1/07	HCH	**−**	**−**
*Mycobacterium marinum*		7091	CECT	**−**	**−**
*Mycobacterium celatum*		342	ATCC	**−**	**−**
*Mycobacterium intracellulare*		CC 2/04	HCH	**−**	**−**
*Mycobacterium simiae*		946	HCH	**−**	**−**
*Mycobacterium smegmatis*		3017	CECT	**−**	**−**
*Mycobacterium flavencens*		3027	CECT	**−**	**−**
*Mycobacterium Phlei*			CECT	**−**	**−**

FMV, Facultad de Medicina Valladolid, Valladolid, Spain; CAJA, Consejeria de Agricultura, Junta de Andalucia, Seville, Spain; CECT, Colección Española de Cultivos Tipo, Valencia, Spain; HCH, Hospital Carlos Haya, Málaga, Spain. ATCC, American Type Culture Collection.

### Clinical sensitivity and specificity

Of the 25 brucellosis patients, diagnosis was established by isolating the pathogen in blood cultures or in cultures of other samples in 17 cases (68%). For the remaining 8 patients (32%), the diagnosis was made by clinical-serological means. All the strains isolated were identified as *B. melitensis*. *Brucella* was isolated in non-blood samples in seven patients (26.9%) (two with vertebral osteomyelitis and one each with meningitis, pleural empyema, liver abscess, knee arthritis and thyroid abscess). M RT-PCR identified *Brucella* DNA in 25 (96.1%) of the 26 samples. The patient with a false-negative M RT-PCR experienced a relapse of knee arthritis and from whom *B. melitensis* was isolated in synovial fluid.

Of the 19 samples obtained from patients with tuberculosis, AFB smears were positive in 9 (52.9%) of the 17 cases in which the test was carried out, culture was positive in 14 (73.7%) cases and M RT-PCR assay was positive in 17 (89.5%) cases. The two negative M RT-PCR results corresponded to one patient with tuberculous vertebral osteomyelitis with a negative culture and another with ankle arthritis from which *M. tuberculosis* was isolated in culture.

M RT-PCR was negative in all the controls, including one with HIV and vertebral osteomyelitis due to *M. xenopi*.

Considering the patients with tuberculosis and brucellosis together, the sensitivity, specificity, PPV and NPV values of our M RT PCR assay were 93.3%, 100%, 100% and 89.7%, respectively, with an accuracy of 95.8% (95% CI, 91.1%–100%) and a negative LR of 0.07 (95% CI, 0.02–0.2) (Table 3).

**Table 3:** Diagnostic yield of Multiplex real-Time PCR in clinical specimens from patients with extrapulmonary tuberculosis and focal complications of brucellosis.

	All samples	Samples from patients with focal Brucellosis	Samples from patients with extrapulmonary TBC
	N = 45	N = 26	N = 19
%, (95% CI)			
**Sensitivity**	93.3, (86–100)	96.2, (88.8–100)	89.5, (75.7–100)
**Specificity**	100	100	100
**PPV**	100	100	100
**NPV**	89.7, (78.6–100)	97.8, (93.6–100)	96.3, (91.3–100)
**Accuracy**	95.8, (91.1–100)	98.6, (95.9–100)	97.2, (93.3–100)
**Positive LR**	ND^a^	ND^a^	ND^a^
**Negative LR**	0.07, 0.02–0.20	0.04, 0.01–0.26	0.11, 0.03–0.39

PPV = positive predictive value; NPV = negative predictive value; Positive LR = positive likelihood ratio, Negative LR = negative likelihood ratio.

a not done for mathematical reasons (division by zero).

**Figure 2:**
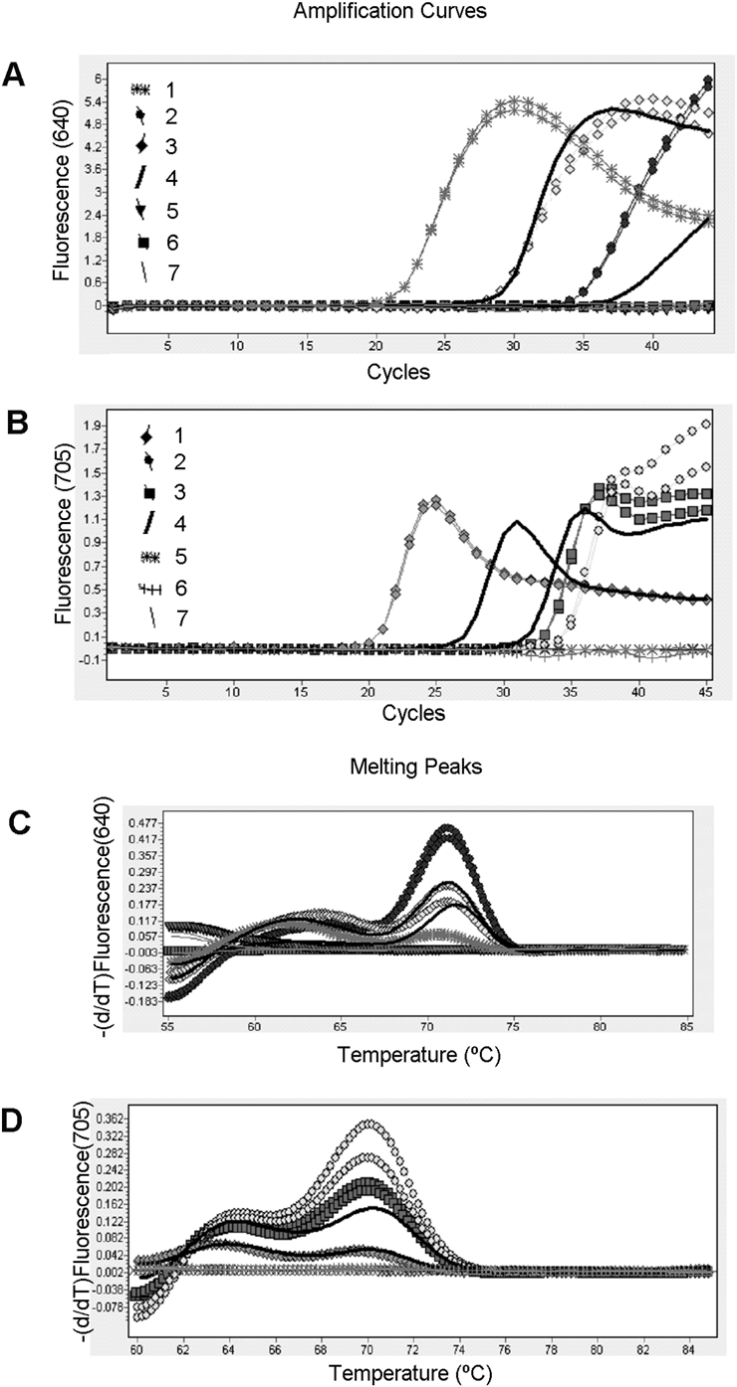
Evaluation of the M RT-PCR assay with clinical samples. A representative set of clinical samples was simultaneously tested for the BCSP31 gene for *Brucella* spp (A) and the intergenic region SenX3-RegX3 for *M. tuberculosis* complex (B). Panel (A) Samples 1, 2 and 3 were pleural fluid, hepatic abscess and urine, respectively, corresponding to brucellosis patients; sample 7 was CSF, from a patient with *S. pneumoniae* meningitis; and samples 4 and 5, positive controls for *Brucella* and *M tuberculosis* complex, respectively. Sample 6, negative control. Panel (B). Samples 1, 2 and 3 were lymph node, pericardial tissue and psoas abscess, respectively, corresponding to tuberculosis patients; sample 7 was of vertebral tissue, from a patient with *S. agalatiae* pyogenic vertebral osteomyelitis; and samples 4 and 5, positive controls for *M. tuberculosis* complex and *Brucella*, respectively. Sample 6, negative control. Panels (C) and (D). Melting curves of the amplified fragments generated by M RT-PCR. Specific signals for brucellosis patients and positive controls had melting temperatures of 71.51±0.18°C and 71.12±0.13°C for tuberculosis patients and positive controls.

The mean Cp was 29.3±4.6 cycles in the patients with *Brucella* and 30.5±5.5 cycles for those with tuberculosis. In both cases melting temperature analysis confirmed the nature of the amplified product (Figure 2).

**Figure 3:**
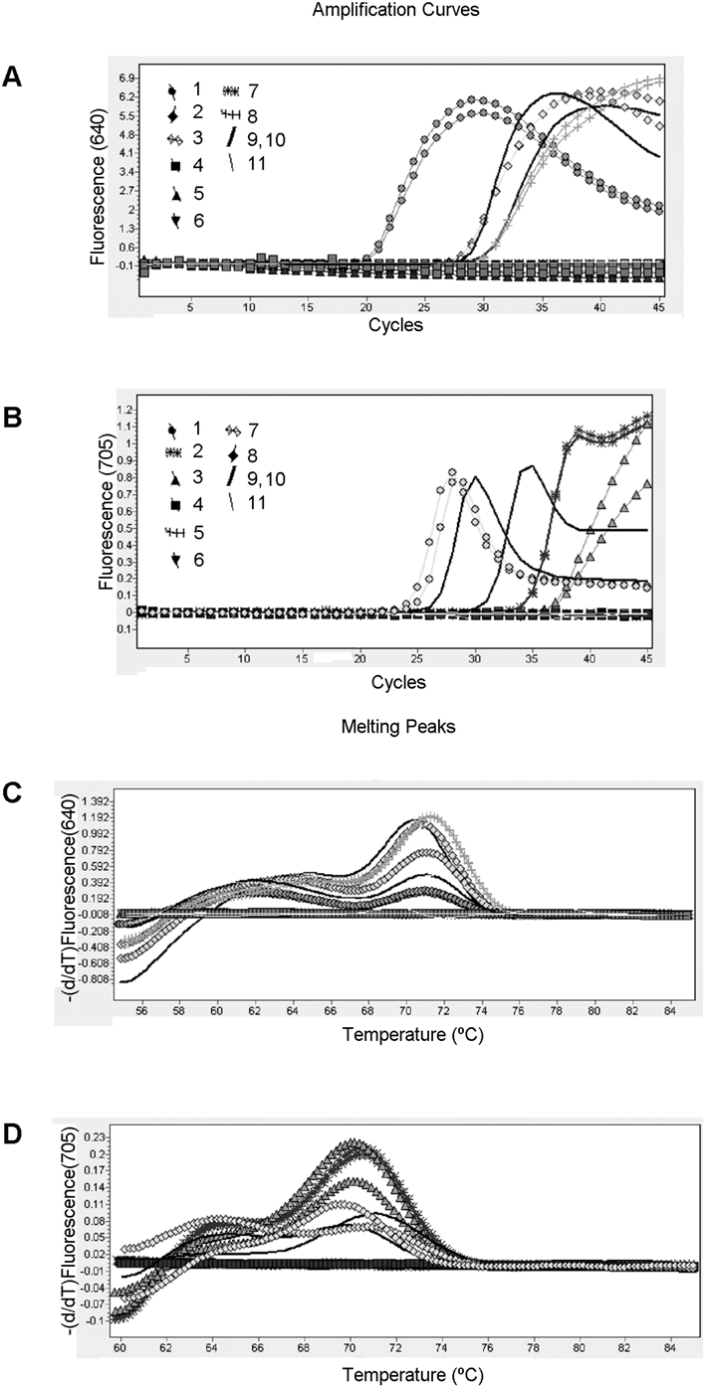
Double-tube format RT-PCR assay with identical clinical samples to those described in Figure 2. All reactions were optimized to obtain the best amplification kinetics under the same cycling conditions and reaction mixture compositions, as described in the Methods section. Panel (A) Clinical samples tested for the BCSP31 gene for *Brucella* spp. Samples 1, 2 and 3 were pleural fluid, hepatic abscess and urine, respectively, corresponding to brucellosis patients. Sample 4 was CSF, from a patient with *S. pneumoniae* meningitis; and samples 5, 6 and 7 were lymph node, pericardial tissue and psoas abscess, respectively, corresponding to tuberculosis patients, and sample 8 was of vertebral tissue from a patient with *S. agalatiae* pyogenic vertebral osteomyelitis. Samples 9 and 10, positive controls for *Brucella*. Sample 11, negative control. Panel (B). Clinical samples assayed for the intergenic region SenX3-RegX3 for *M. tuberculosis* complex, Samples 1 to 8 were identical to those described in panel A. Samples 9 and 10, positive controls for *M. tuberculosis* complex. Sample 11, negative control. Panels (C) and (D). Melting curves of the amplified fragments generated by RT-PCR in double-tube format.

The M RT-PCR results were similar when an identical panel of samples was assayed in a double-tube format (Figure 3).

## Discussion

Traditional laboratory techniques for the diagnosis of tuberculosis and brucellosis are far from being sensitive and specific. In the case of tuberculosis, direct microscopy lacks sensitivity and serological diagnosis of brucellosis lacks adequate specificity. Moreover, in both cases, cultures are time consuming and require direct sample handling, representing a risk of infection for laboratory personnel.

PCR has revolutionized the field of infectious disease diagnosis. Molecular techniques have proven more sensitive than conventional methods in both tuberculosis and human brucellosis [10]–[11], [17]–[20]. Real-time PCR technology has provided an opportunity to develop an assay that meets the requirements for rapid diagnosis, thus increasing the interest of clinical laboratories in molecular diagnosis [21].

Multiplex polymerase chain reaction is a variant of PCR in which two or more target sequences can be simultaneously amplified. Multiplex PCR has the potential to produce considerable savings in time and effort in the laboratory. This method has been successfully applied in many areas of DNA testing, including the field of infectious diseases [22]. From a clinical and microbiological point of view, multiplex PCR would be specially useful in those scenarios in which several agents cause similar clinical syndromes.

Numerous PCR assays employing a number of different *M. tuberculosis* and *Brucella* spp targets have recently been described [23]–[24]. For the detection of *Brucella*, we chose a conserved region of the gene encoding for BCSP31, the target with the most clinical experience [11], [25]–[26]. For tuberculosis, we opted for specificity. The IS*6110* multicopy insertion element, the most widely used target sequence of the *M. tuberculosis* genome, does not allow specific identification of *M. tuberculosis* [27]. Moreover, *M. tuberculosis* strains lacking the IS*6110* element have been described [28]. Accordingly, we selected a sequence of the SenX3-RegX3 intergenic region (IR) which contains mycobacterial interspersed repetitive units (MIRUs), described only for mycobacterial species belonging to the *M. tuberculosis* complex [29]–[30].

Our results demonstrate the specificity of M RT-PCR. All the strains of *Brucella* spp and *M. tuberculosis* complex showed clear DNA amplification, confirmed by melting curve analysis and sequencing the amplified products, which perfectly matched the 207 bp and 164 bp fragments corresponding to *B. abortus* and the SenX3-RegX3 intergenic region of *M. tuberculosis* complex. No cross-reaction occurred with any of the strains of the wide panel of non-tuberculous micobacteria tested. These results agree with those of Broccolo et al [29] and appear to confirm that the amplification of a DNA fragment belonging to the SenX3-RegX3 IR is very specific and allows for a more precise identification of the *M. tuberculosis* complex species.

Under the conditions used, the precision of our M RT-PCR can be considered very high, as the intra-assay variation was lower than 1% and the inter-assay variation around 2%. Bearing in mind that the inoculum found in clinical samples from patients with extrapulmonary tuberculosis or focal complications of brucellosis could be very small, the detection capacity of any multiplex PCR assay used in these diagnoses needs to be very high. The analytical sensitivity of our M RT-PCR, 2 genome equivalents, can also be considered very good, as it is similar or higher than that of conventional PCR procedures previously used for the diagnosis of tuberculosis or brucellosis. Such small amounts of DNA can be expected in any clinical sample from a patient with active extrapulmonary tuberculosis or focal brucellosis.

Numerous studies have assessed the yield of PCR techniques for the diagnosis of extrapulmonary tuberculosis [10], [18]–[20] and a few others for focal complications of brucellosis [11], [17]. Nevertheless, this is the first study designed to verify the usefulness of multiplex technology applied to the rapid differential diagnosis between extrapulmonary tuberculosis and focal complications of brucellosis. Overall, the sensitivity and specificity of our M RT-PCR were very high, correctly identifying 93.3% of the patients with tuberculosis or brucellosis, and showing negative in all the controls. Individually, the sensitivity in the diagnosis of patients with focal complications of brucellosis was 96.2%, similar to that reported for an in-house PCR assay [11] and far better than culture, for which positivity was again shown to be below 50%. The sensitivity in clinical specimens of extrapulmonary tuberculosis was slightly lower, 89.5%. Even so, this was still higher than most studies, which have used different targets and different amplification strategies and reported sensitivity values of the various PCR techniques from 53.8% to 85% [10], [18]–[20].

Apart from its high sensitivity, M RT-PCR provides the results in just four hours, far less than that required for isolation of *Brucella* and *M. tuberculosis*. The mean time for isolation and identification of *Brucella* in a non-blood sample was 5.8±2.6 days and for *M. tuberculosis* it was 21.5±6.8 and 9.5±4.4 days, depending on whether solid or liquid media were used, respectively. Even assuming that culture is the gold standard for diagnosis of tuberculosis and that it is the only method enabling a study of strain sensitivity to treatment, this drastic reduction in diagnostic delay has important prognostic implications in severe cases, such as meningitis or vertebral osteomyelitis.

Finally, like other molecular techniques, M RT-PCR is very versatile, as samples can be stored at −20°C until processing and it almost completely obviates the need for direct handling of the pathogens, thus drastically reducing the risk of infection of laboratory personnel.

One limitation of this study is the reduced sample size and the diversity of the samples used. Caution should therefore be exercised with definitive interpretation of the results. Nevertheless, this is a frequent problem with diseases with a relatively low incidence and whose form of presentation is very heterogeneous.

In conclusion, under the conditions used here, M RT-PCR seems to be sensitive and specific, which, coupled with its speed and versatility, make this technique a very useful tool for the differential diagnosis between extrapulmonary tuberculosis and certain focal complications of brucellosis.
