# Correction: Rapid Differential Diagnosis between Extrapulmonary Tuberculosis and Focal Complications of Brucellosis Using a Multiplex Real-Time PCR Assay

**DOI:** 10.1371/annotation/ea7005a7-92fe-4091-a9d0-d9a061a05a59

**Published:** 2009-02-24

**Authors:** María Isabel Queipo-Ortuño, Juan D. Colmenero, Pilar Bermudez, María José Bravo, Pilar Morata

The fifth author, Pilar Morata, was listed incorrectly as affiliated with both #1 and #2, Dr. Morata should only be affiliated with #1: Biochemistry and Molecular Biology Department, Faculty of Medicine, University of Malaga, Malaga, Spain.

